# Non-dysraphic Thoracic Spinal Lipoma in an Obese Adult Woman: A Case Report

**DOI:** 10.7759/cureus.98832

**Published:** 2025-12-09

**Authors:** Mitsuyoshi Abe, Yuki Sakaeyama, Ryo Matsuzaki, Shuhei Kubota, Nobuo Sugo

**Affiliations:** 1 Neurosurgery, Toho University, Tokyo, JPN

**Keywords:** non-dysraphic type, obesity, spinal lipoma, spinal tumor, thoracic spine

## Abstract

Spinal lipoma is a rare benign tumor characterized by abnormal proliferation of mature adipose tissue within the spinal canal. The non-dysraphic type typically occurs in adults without vertebral anomalies and is extremely rare at the thoracic level. We report a case that developed in the setting of severe obesity, suggesting a possible link between obesity and tumor progression. A 51-year-old woman (152.2 cm, 81.3 kg, BMI 35.1) with a history of diabetes and prior lumbar disc surgery resulting in cauda equina injury presented with progressive right-dominant periumbilical dysesthesia for two years. Neurological examination revealed right-sided hypoalgesia at the Th7-L1 dermatomes. Thoracic MRI revealed a well-demarcated intradural extramedullary mass on the right side at the Th8-9 level, showing hyperintensity on both T1- and T2-weighted images and hypointensity on fat-suppressed sequences, consistent with a spinal lipoma. CT demonstrated no bony abnormalities or evidence of spinal dysraphism. Under general anesthesia, a wide laminectomy at Th7-8 and dome laminectomies at Th6 and Th9 were performed. A yellowish, soft fatty mass was found firmly adherent to the spinal nerve roots. A partial resection was performed for decompression, leaving a small portion of fatty tissue caudally. Histopathological examination confirmed lipoma. Postoperatively, the patient’s truncal dysesthesia entirely resolved with no new neurological deficits; bladder and rectal dysfunction remained unchanged. No recurrence was observed on MRI during the one-year follow-up. This rare case of non-dysraphic thoracic spinal lipoma occurred in the setting of severe obesity. Obesity and weight fluctuations may have contributed to tumor growth and symptom progression; partial decompression with long-term MRI follow-up is recommended.

## Introduction

Spinal lipoma is a rare benign tumor characterized by the abnormal proliferation of mature adipose tissue within the spinal canal, accounting for less than 1% of all spinal cord tumors [1]. These lesions are broadly classified into dysraphic and non-dysraphic types. Dysraphic lipomas are typically associated with neural tube closure defects such as spina bifida and usually present in childhood. In contrast, non-dysraphic spinal lipomas occur in the absence of vertebral anomalies and are generally diagnosed in adulthood [2,3]. Their embryological origin is thought to involve aberrant inclusion of ectodermal or mesenchymal elements during neurulation, distinguishing them from dysraphic variants.

Non-dysraphic lipomas have been reported at various levels of the spinal axis, including the cervical, thoracic, and thoracolumbar regions; however, thoracic involvement remains particularly uncommon [3,4]. Although rare, several adult thoracic cases have been documented, underscoring the importance of recognizing this entity in clinical practice.

Recent literature has suggested that obesity and metabolic abnormalities may influence the behavior of spinal lipomas, including potential enlargement or symptom progression [5]. However, these associations remain speculative, and causality has not been established. In this context, our case adds to the limited number of thoracic non-dysraphic lipomas in adults. It highlights the potential, though not definitive, role of obesity as a contributing factor.

We report a rare case of a thoracic non-dysraphic spinal lipoma in an adult woman with severe obesity and discuss the radiological, surgical, and clinical features in relation to existing literature.

## Case presentation

**Figure 1:**
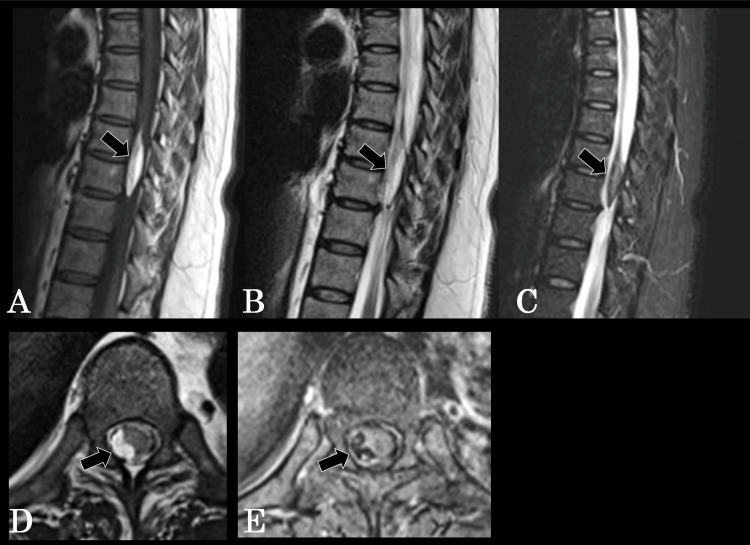
Thoracic spine MRI (A) T1-weighted image, (B) T2-weighted image, and (C) fat-suppressed image. (D) Axial T2-weighted image demonstrating a right-sided intradural extramedullary lipoma compressing the spinal cord leftward. The tumor area measures 39.51 mm², within a subarachnoid space of 114.58 mm², representing 34.5% occupancy of the available intradural space. (E) Axial fat-suppressed image showing complete signal suppression of the lesion, confirming its fat composition and depicting its relationship to the displaced spinal cord and adjacent nerve roots. MRI: magnetic resonance imaging

The patient was a 51-year-old woman, 152.2 cm tall and weighing 81.3 kg, with a BMI of 35.1, indicating severe obesity. Her past medical history included diabetes mellitus. Sixteen years earlier, she had undergone lumbar disc herniation surgery at the L5-S1 level at another hospital. Following the surgery, she developed bladder and rectal dysfunction due to cauda equina injury and had continued intermittent self-catheterization thereafter. Two years prior to presentation, she began experiencing dysesthesia around the periumbilical region, predominantly on the right side. These symptoms had recurred two to three times per year, with each episode resolving spontaneously within about two weeks. Thoracic MRI revealed a well-demarcated intradural extramedullary mass located on the right side of the spinal cord at the Th8-9 level. The lesion appeared hyperintense on both T1- and T2-weighted images and hypointense on fat-suppressed images, suggesting a spinal lipoma (Figure 1A-1C). Approximately one month before the presentation, the symptoms persisted and gradually worsened, prompting her to visit our department of neurosurgery for further evaluation. At presentation, the patient was alert and fully oriented, with no abnormalities in cranial nerves I-XII. Manual muscle testing (MMT) results are shown in Table 1.

**Table 1:** Summary of MMT Mild weakness was observed in the right iliopsoas, quadriceps, and hamstrings compared with the normal reference range (5/5). MMT: manual muscle testing

Muscle (MMT)	Right side	Left side	Reference range (normal)	Findings/comments
Iliopsoas	4+/5	5/5	5/5	Mild weakness on the right side
Gluteus maximus	5/5	5/5	5/5	Normal
Quadriceps	4+/5	5/5	5/5	Mild weakness on the right side
Hamstrings	4+/5	5/5	5/5	Mild weakness on right side
Tibialis anterior	5/5	5/5	5/5	Normal
Gastrocnemius	5/5	5/5	5/5	Normal

Pathological reflexes, including Babinski and Chaddock signs, were negative bilaterally. Gait was normal and stable. Sensory examination revealed right-dominant hypalgesia and paresthesia at the Th7-L1 dermatomal levels, while tactile and thermal sensations were preserved. No cutaneous abnormalities, such as hair tufts, dimples, or nevi, were observed along the midline of the back.

Thoracic MRI revealed a well-demarcated intradural extramedullary mass located on the right side of the spinal cord at the Th8-9 level. The lesion appeared hyperintense on both T1- and T2-weighted images and hypointense on fat-suppressed sequences, consistent with a spinal lipoma (Figure 1A-1C). Axial MRI at the level of maximal compression demonstrated a right-sided intradural extramedullary mass measuring 39.51 mm² within a subarachnoid space of 114.58 mm², corresponding to 34.5% of the intradural area and causing leftward displacement of the spinal cord. The lesion was hyperintense on T2-weighted images and showed complete signal loss on fat-suppressed images, confirming its fat content and delineating the degree of spinal cord compression (Figure 1D-1E).

**Figure 2:**
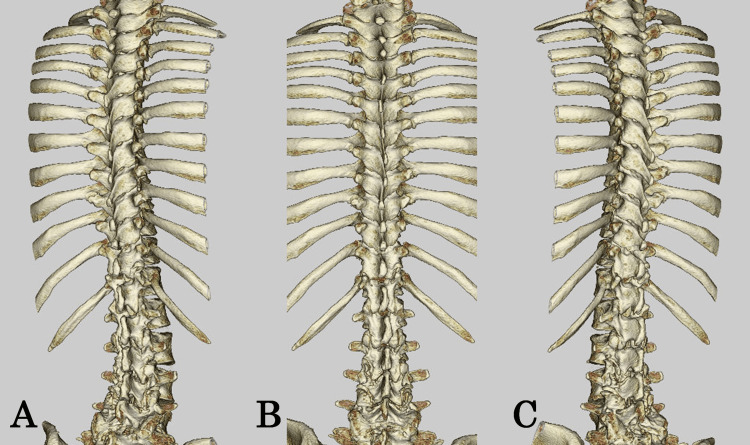
Thoracic spine CT (A) Right posterior oblique view, (B) posterior view, (C) and left posterior oblique view. No bony abnormalities or evidence of spinal dysraphism were observed. CT: computed tomography

Thoracic CT demonstrated no bony abnormalities or evidence of spinal dysraphism (Figure 2A-2C). No radiological findings suggestive of a tethered cord were observed. The conus was normally positioned, and there was no evidence of abnormal tension or associated dysraphic changes.

**Figure 3:**
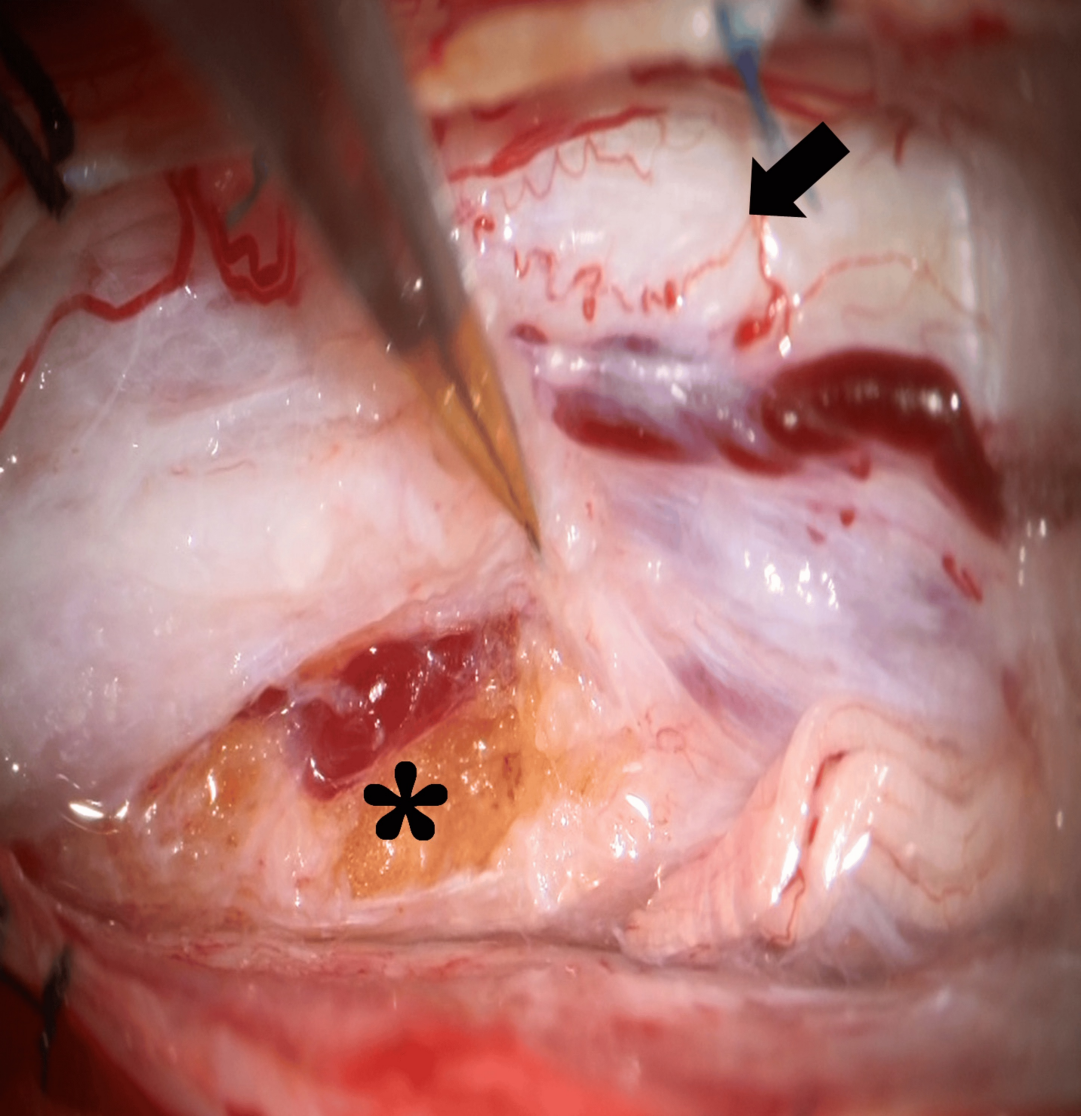
Intraoperative photograph Arrow: spinal cord, asterisk: lipoma. A yellowish, soft, fatty mass was observed beneath the dura mater, firmly adherent to the nerve roots.

Under general anesthesia, tumor resection was performed. A wide laminectomy was carried out at Th7-8, and dome laminectomies were performed at Th6 and Th9, followed by a midline dural incision. Intraoperatively, a yellowish, soft, fatty mass was observed, firmly adherent to the spinal nerve roots (Figure 3).

**Figure 4:**
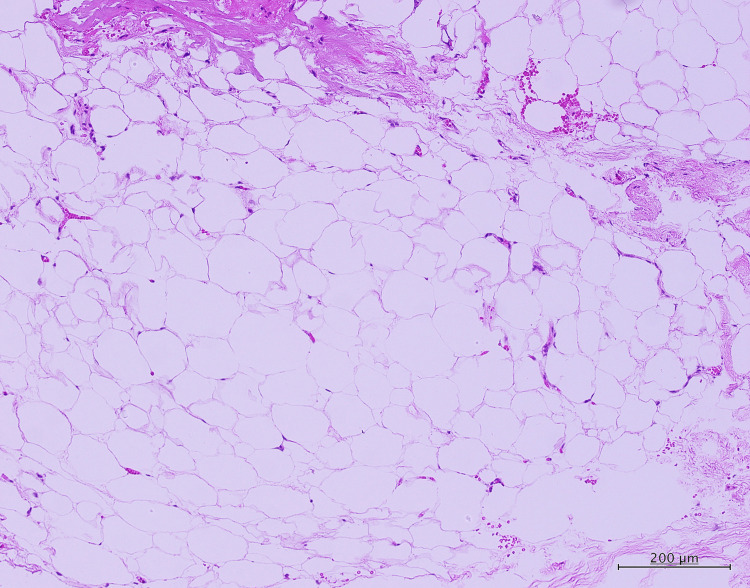
Histopathological findings The lesion consisted of mature adipocytes without atypia and interspersed collagen fibers, consistent with a diagnosis of lipoma.

Careful dissection was performed to preserve the nerve roots, and the tumor was removed as extensively as possible. Because the border between the tumor and spinal cord parenchyma was indistinct, a small portion of the fatty tissue was intentionally left caudally to prevent neurological injury. Histopathological examination revealed mature adipocytes without atypia and interspersed collagen fibers, consistent with a diagnosis of lipoma (Figure 4).

The postoperative course was uneventful. The abnormal periumbilical sensations resolved completely, and no new motor or sensory deficits developed. Bladder and rectal dysfunction remained unchanged from the preoperative state. No recurrence was observed during one year of postoperative follow-up. Written informed consent was obtained from the patient for publication of this case report and accompanying images.

## Discussion

The etiology of non-dysraphic spinal lipoma remains uncertain, but it is believed to originate from abnormalities in neural tube formation (neurulation) during embryonic development [6,7]. Premature disjunction of the surface ectoderm during primary neurulation may allow ectodermal or mesenchymal elements to become entrapped within the closing neural tube, where they subsequently differentiate into adipocytes [7]. Similarly, lesions involving the filum terminale are thought to arise from abnormal aggregation of caudal cell masses during secondary neurulation, leading to incorporation of adipose tissue into neural structures [7]. Thus, non-dysraphic spinal lipomas are considered hamartomatous malformations rather than true neoplastic lesions.

The etiology of non-dysraphic spinal lipomas remains uncertain, but they are generally considered congenital hamartomatous malformations arising from aberrant inclusion of ectodermal or mesenchymal elements during neurulation [7,8]. In adults, clinical progression can vary, and the factors influencing symptom onset or enlargement are not fully understood.

Several reports have speculated that obesity or metabolic abnormalities might influence the behavior of spinal lipomas, including possible enlargement or symptom progression [5]. However, these associations are not supported by longitudinal measurements or objective growth data. In our case, no serial imaging or body weight records were available, and therefore, a causal relationship between obesity and tumor behavior cannot be established. The potential mechanisms described in previous studies, such as adipocyte hypertrophy, inflammatory signaling, or altered lipid metabolism, should be regarded as hypotheses rather than explanations applicable to this single case.

Our contribution to the existing literature includes a detailed radiological assessment with quantitative axial MRI measurements, demonstrating the degree of spinal cord compression and confirming the lesion's fat composition. These findings help clarify the anatomical rationale for surgical decision-making, particularly in thoracic nondysraphic lipomas, which are rarely reported.

Given that a portion of the lesion was intentionally left in situ, long-term follow-up is essential. The present one-year follow-up is relatively short, and the absence of additional imaging data represents a limitation. Future evaluations may provide further insight into postoperative stability, symptom recurrence, or potential regrowth.

Spinal lipomas are often tightly adherent to the spinal cord and nerve roots, and total resection carries a significant risk of neurological injury [3,9]. Therefore, many reports recommend partial resection aimed at decompression as a safer approach. In our case, due to firm adhesion between the tumor and the nerve roots, a small amount of fatty tissue was intentionally left caudally. The postoperative outcome was favorable, with complete resolution of sensory symptoms and no new deficits. Previous studies have shown that neurological outcomes after partial removal are generally good, and recurrence is rare [2].

## Conclusions

We presented a rare case of a non-dysraphic spinal lipoma located at the thoracic level. This entity should also be considered in the differential diagnosis when evaluating intradural extramedullary lesions, particularly those demonstrating fat-intensity signals on MRI. Although the patient had severe obesity, our data do not include longitudinal body weight information or serial imaging measurements; therefore, no causal relationship between obesity and tumor enlargement can be established in this case. Any potential association should be interpreted as hypothetical.

This case contributes to the literature by providing detailed radiological characterization, including quantitative axial MRI measurements that clarify the degree of spinal cord compression and support the rationale for surgical planning. Because a portion of the tumor was intentionally left in situ and the follow-up period was limited to one year, longer postoperative surveillance is necessary to assess long-term stability and the possibility of regrowth. These limitations should be considered when interpreting the clinical implications of this report.
